# Predicting Free-Space Occupancy on Novel Artificial Structures by an Invasive Intertidal Barnacle Using a Removal Experiment

**DOI:** 10.1371/journal.pone.0074457

**Published:** 2013-09-04

**Authors:** Sally A. Bracewell, Leonie A. Robinson, Louise B. Firth, Antony M. Knights

**Affiliations:** 1 School of Environmental Sciences, University of Liverpool, Liverpool, United Kingdom; 2 Ryan Institute, National University of Ireland Galway, Galway, Republic of Ireland; University of Waikato (National Institute of Water and Atmospheric Research), New Zealand

## Abstract

Artificial structures can create novel habitat in the marine environment that has been associated with the spread of invasive species. They are often located in areas of high disturbance and can vary significantly in the area of free space provided for settlement of marine organisms. Whilst correlation between the amount of free space available and recruitment success has been shown in populations of several marine benthic organisms, there has been relatively little focus on invasive species, a group with the potential to reproduce in vast numbers and colonise habitats rapidly. Invasion success following different scales of disturbance was examined in the invasive acorn barnacle, *Austrominius* *modestus* , on a unique art installation located in Liverpool Bay. Population growth and recruitment success were examined by comparing recruitment rates within disturbance clearings of 4 different sizes and by contrasting population development with early recruitment rates over a 10 week period. Disturbed areas were rapidly recolonised and monocultures of *A* *. modestus* formed within 6 weeks. The size of patch created during disturbance had no effect on the rate of recruitment, while a linear relationship between recruit density and patch size was observed. Density-dependent processes mediated initial high recruitment resulting in population stability after 8-10 weeks, but densities continued to greatly exceed those reported in natural habitats. Given that artificial structures are likely to continue to proliferate in light of climate change projections, free-space is likely to become more available more frequently in the future supporting the expansion of fast-colonising species.

## Introduction

The introduction of novel species into vulnerable habitats can impact upon the balance of species and allocation of resources. Although chance and timing play an important role [1], invasion success is often attributed to biological traits such as high fecundity, short generation times and environmental tolerance, which allow introduced species to colonise new or disturbed habitats quickly [2,3]. In marine benthic communities, such as intertidal rocky shores, naturally occurring or anthropogenic disturbance can create free-space and reset successional processes [4], opening the door to non-native species and facilitating their establishment [5]. The creation of free-space by the introduction of artificial structures can have a similar effect.

The colonisation of natural habitats by an invasive species following disturbance can be offset by the presence of an established native biota [6], such that the invader occurs in limited numbers and fails to become established in meaningful abundances [7]. In contrast, newly installed artificial structures are depauperate of species, and can become quickly dominated by a limited number of species [8,9] due to features such as reduced habitat heterogeneity [9,10] or increased refugia from predators [11,12]. Artificial structures are rapidly becoming ubiquitous features of the marine environment due to urbanisation and proliferation of both artificial coastal defences and renewable energy schemes [13,14], introducing large expanses of hard substrata in areas that are otherwise sedimentary, often at the expense of natural habitats [14–16].

A primary factor in determining which species will colonise an artificial structure is its placement, and specifically the proximity (connectivity) of the structure to a source population (i.e. a rescue effect). Many marine species exhibit a planktonic larval stage [17], facilitating their dispersal away from their natal patch. Dispersal distance can be described using a combination of physical and biotic processes such as ocean circulation [18], near shore oceanography, wind [19] and planktonic duration [20]. However, spatial and temporal variation in propagule pressure [21] and multiple post-settlement processes [22] such as rate of supply [23], the timing of recruitment [1,24] and availability of resources [25] can confound outcomes such that even small-scale location differences can affect recruitment success.

Artificial structures are implicated in the spread of invasive and non-native species by acting as stepping stones to dispersal across areas of unsuitable habitat (i.e. sedimentary substrata) [26]. Morphological features of the structure can affect the type of species that are able to colonise. Construction materials can range from small cobbles to large boulders or concrete pilings [10] resulting in patches of habitat of different size and shape [12,16,27], which can affect the emergent population or community structure [9,28]. Predicting the recruitment of species to those structures and, specifically, estimating their population size is an important step in determining if a structure has the potential to act as a viable source population that can facilitate spread. If the area of habitat is too small, the population may fail to persist at that location [29], or the adult population will generate too few offspring such that its spread is unviable [30,31]. If the area of habitat is too large, there may be insufficient resources to support the population [25] or insufficient habitat heterogeneity to provide refugia from predation and/or abiotic stress [11,32].

*Austrominius* *modestus* (Darwin, 1854) has become widespread along UK coasts since its introduction in the late 1940s [33] and exhibits traits typical of invasive species [2]. Abundant on rocky shores throughout Europe [34], *A* *. modestus* can compete with native species of barnacle including *Semibalanusbalanoides* and *Balanusimprovisus* [34] but can also form monocultures on artificial structures [27]. One such artificial structure is the art installation, ‘Another Place’, situated on the west coast of the United Kingdom. Using this unique model system, we created disturbances of different magnitudes to test the hypothesis that small increases in initial free-space would influence the recruitment of *Austrominius* *modestus* and additionally, that these changes would affect the success and density of recruits over time. Rate of recruitment to free space plots was monitored every 2 wk over a 10 wk period and predictions of population development and growth were generated.

## Methods

### Ethics Statement

No permits or ethics approval were required for the described study, nor did it involve any endangered or protected species. The sampling of organisms was arranged jointly through the University of Liverpool and Sefton Council and complied with all relevant regulations.

### Study site

*Austrominius* *modestus* has been present in the Liverpool Bay area since the 1950s [34] and is found in abundance on the Antony Gormley art installation ‘Another Place’ at Crosby Beach, Liverpoool (for images and detailed map please see 27). Crosby Beach is a sheltered, south-westerly facing beach stretching 5 km north-west from the Port of Liverpool to the River Alt. The beach is characterised by semi-diurnal tides with a spring tidal range in excess of 10 m. The artificial structures consist of 100 replicate cast-iron life-size human figures spread over approximately 3 km on an otherwise sandy beach. The 100 structures (each 191 cm in height) are positioned over a range of tidal heights from low to high shore and extend approximately 1 km out to sea. A year after they were installed in July 2005 *A* *. modestus* was the dominant species present [27]. Today, the majority of statues are covered almost entirely by *A* *. modestus* (Bracewell et al. *pers. obs.*).

### Austrominius modestus recruitment rates

The study ran for 10 weeks between late May and August 2011 and coincided with peak planktonic abundances, although recruitment can occur year-round [35]. At the start of the experiment, a series of free-space plots were created within the adult *A* *. modestus* population on the torso region of 10 statues. Statues were randomly chosen from those located in the mid-shore region where recruitment is consistently high [27]. The torso region was chosen due to its uniform surface and the fact that it was of sufficiently large size to allow multiple independent plots to be established. Four square, free-space plots of different size (area) were created on each statue by removing all barnacles from the predefined area using a masonry chisel. Plot sizes were 25 cm^2^, 19 cm^2^, 12 cm^2^ and 6 cm^2^ respectively. Each plot was then left undisturbed for the duration of the study and photographed every 2 wk for a total of 10 wk (when plots were filled) using a 12-megapixel digital camera (Ricoh G600, Japan) with a fixed lens aperture (using the macro setting) mounted to a metal frame with an integrated 5 x 5 cm quadrat for scale. The total number of individuals in each plot (all individuals, including those settled on top of each other, were counted as recruits) were enumerated using the count tool in the freeware image analysis program *ImageJ* [36] (NB The average age of maturation of *A* *. modestus* can vary from between 10–15 wk post-settlement depending on the surrounding temperature [37]. Therefore, maturation could not occur before the end of the experiment and thus any individual within a plot is by definition a recruit). We refer to these plots as the ‘cumulative’ plots herein.

### Control Plots

An additional plot of 25 cm^2^ was created on each statue torso that was photographed every 2 wk and then re-cleared (i.e. disturbed every 2 wk) for the duration of the study. We refer to this additional plot as the ‘control plot’ which acts as a proxy for early recruitment based on the assumption that no density-dependent or density-independent processes occur during this time [38].

#### Data Analysis

A linear mixed-effects model (nlme package) [39] was used to compare changes in recruit abundance in plots over time. Model factors were: (1) Initial Cumulative Plot Size (25, 19, 12, 6 cm^2^) (2), Time (2, 4, 6, 8, 10 wk) as fixed effects and (3) Statue as a random grouping factor. A first-order autoregressive (AR(1)) correlation structure was used to account for the repeated measures on the same plot on a statue. The significance of the fixed effects was analysed using the likelihood ratio test based on the maximum likelihood (ML) estimation procedure [39]. Linear contrasts were used for post hoc comparisons. Data were square root transformed to fulfill the requirements of the statistical analysis for normal distribution and homogeneity of variance.

Change in recruit abundance among plots of different size was compared in two ways. First, using the total recruit abundance irrespective of plot size, and second, using recruit abundance standardised by the initial plot size (i.e. abundance per unit area) to test if recruitment rate varied in response to the amount of free-space created.

## Results

### Temporal changes in control and cumulative recruitment in 25 cm^2^ plots

**Figure 1:**
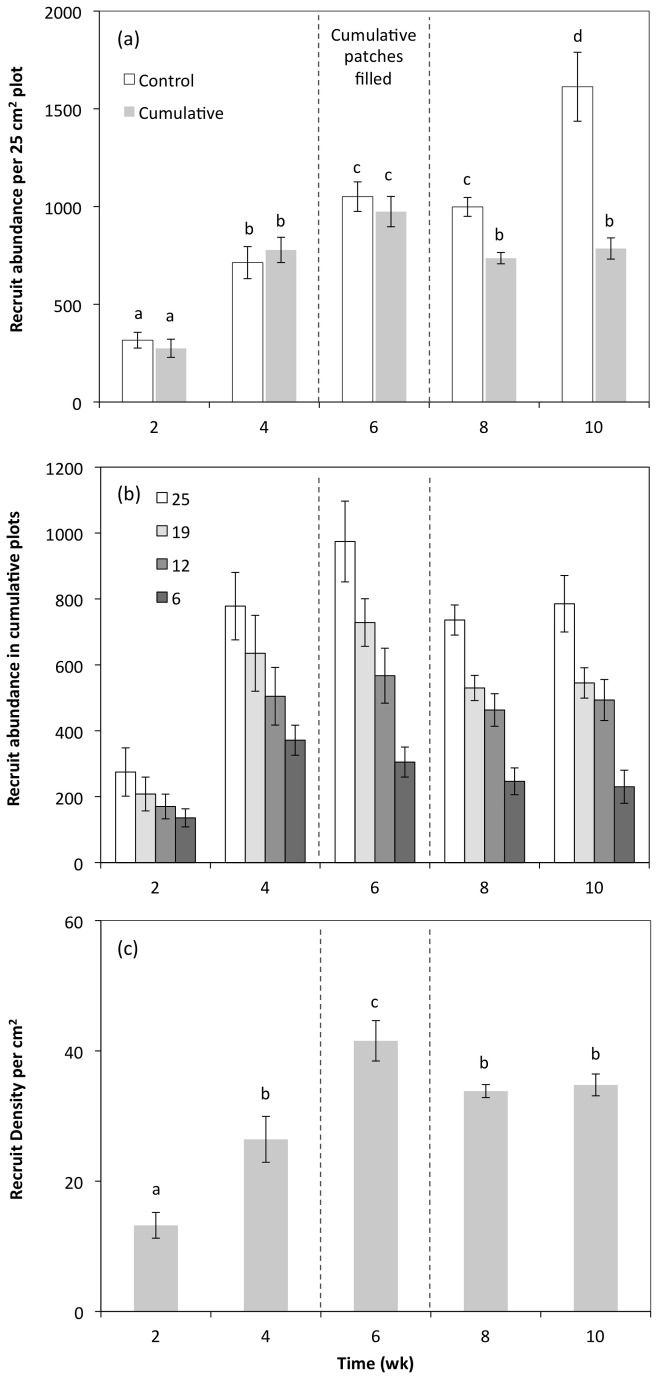
Recruit abundances of *Austrominius modestus* over time. (a) Mean recruit abundance (± SE) in 25 cm^2^ control and cumulative plots every 2 wk (*n* = 10), (b) mean recruit abundance (± SE) in plots of difference area (cm^2^) (*n* = 10), and (c) mean recruit density per cm^2^ in cumulative plots in plots of different sizes (*n* = 40). Letters over bars (a–d) indicate post-hoc comparison outcomes with the same letter indicating no significant difference between recruit abundance/density. All plots irrespective of initial clearance size are pooled in (b). The period when all cumulative plots were 100% colonised is shown (dotted line).

**Figure 2:**
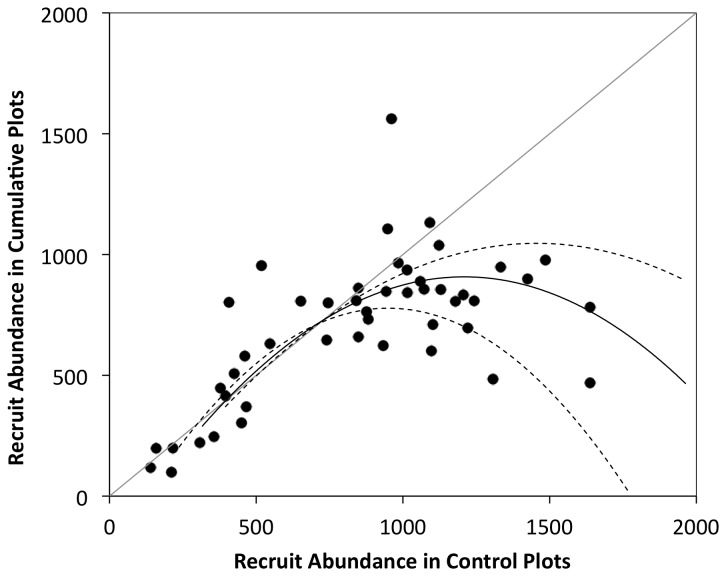
Comparative recruit density in cumulative and control plots. Change in recruit abundance in 25 cm^2^ control and cumulative plots. Recruitment was estimated every 2 wk using non-destructive photographic sampling and image analysis. Control plots were disturbed (cleared) every 2 wk and estimates are compared to its paired cumulative plot on the same statue. Cumulative plots were undisturbed following the initial clearance. Significant regression is shown (solid line; *R* ^2^ = 0.89) and dashed lines indicate 95% confidence intervals. 45^o^ line indicates the point where recruitment is the same in control and cumulative plots.

*Austrominius* *modestus* recruits were recorded on all statues in all plots at all times over the 10 wk but their abundance varied greatly over time and among plots. In control plots, there was significant variation in recruitment over time, increasing week-on-week (with the exception of between wk 6 and 8 where no significant difference was found) from 316 ± 40 per 25 cm^2^ in wk-2 and increasing five-fold to a maximum of 1613 ± 176 per 25 cm^2^ in wk-10 (*F*_*4,45*_ = 32.45, p < 0.001). The density of *A* *. modestus* in 25 cm^2^ cumulative plots increased in line with the bi-weekly recruitment in control plots for the first 6 wk, after which there was a significant decline in recruit density in comparison to the control plots (Figure 1a). Maximum density in 25 cm^2^ cumulative plots reached 974 ± 78 recruits in wk-6 before declining to comparable average densities of 736 ± 29 and 785 ± 54 recruits per 25 cm^2^ in wk-8 and 10 respectively. The decline in recruit density in cumulative plots in comparison to recruitment to control plots was best described by a non-linear 2^nd^ order polynomial model (R^2^ = 0.89, p < 0.01) (Figure 2).

### Recruitment into cumulative plots of different size

The abundance of *A* *. modestus* in cumulative plots changed over time and in response to plots of different size (F_12,180_ = 3.86, p < 0.0001) (Figure 1b). At each time point, recruit abundance was greatest in the largest (25 cm^2^) plots and fewest were recorded in the smallest (6 cm^2^) plots. There was no significant difference in recruit abundance in the 12 and 19 cm^2^ plots, although trends suggest increased abundances in the larger of the two plot sizes.

Converting abundance data to density estimates (abundance per unit area) revealed no significant difference in recruitment densities between plots of different size over time (F_12,180_ = 1.21, p = 0.28), nor a significant effect from changing the plot size (F_3,180_ = 1.82, p = 0.15). However, there was a significant difference in recruit density over time (F_4,180_ = 40.0, p < 0.0001) as free-space plots became colonised by *A* *. modestus* . Recruit densities were lowest after 2-wk (~13 ± 1.9 recruits per cm^2^) then doubled to 26 ± 3.5 per cm^2^ after wk-4 followed by a 60% increase in wk-6, the point at which all free-space was filled in all plots (Figure 1c). After wk-6, recruit densities then declined as individuals grew and stabilised around 34.3 recruits per cm^2^ with no significant differences in density between wk 8 and 10 (Figure 1c).

## Discussion

We tested the ability of an invasive species to colonise artificial habitat following the removal of adults and creation of free-space. Comparisons of control and cumulative plots of the same size revealed differences in recruitment rate dependent on the pre-existence of juvenile conspecifics within a plot and remaining free-space. While recruit abundance changed over time, firstly increasing until all free-space became occupied after 6 wk followed by a reduction in recruit abundance after 8 to 10 wk to a stable density, there was no change in recruit density with changes in plot size indicating that recruitment was not limited in this area [40]. This suggests that in other non-recruitment limited areas, a similar growth model could be used to predict the recruitment of *A* *. modestus* on newly installed artificial structures.

Gaines and Roughgarden [41] suggested that space is only important when recruitment is limited and that when the supply of larvae is saturating (non-limited), recruitment is simply a function of the amount of available space [42]. Given that space alone could be used to predict *A* *. modestus* density, this suggests that larval supply is not limited (sensu [40]). The high reproductive output of an adult *A* *. modestus* and annual presence within the plankton [35] was reflected in the high rate of recruitment and rapid colonisation of free-space within 6 wk of clearance. Recruit density was initially high and all free space was filled quickly before the number of recruits declined. Post-settlement mortality can be controlled by density-dependent processes [38,43,44] and thus by changes in propagule pressure [38,45]. After 6 wk, all available space was filled and recruit densities exceeded 40 individuals cm^-2^. Recruits grew rapidly, but after 8 wk, mortality was observed in extant individuals, which was attributed to excessive crowding (Bracewell, pers. obs.) and is indicative of the availability of resources (carrying capacity) within the habitat. Competition for resources including space is a common source of mortality for recruits [38] and some sessile species can reduce mortality from space-limits by displaying morphological plasticity [46], but when progeny vastly outnumber available space, some mortality is inevitable. Maximum population size (assuming densities can be supported and food is not limited) will most likely be determined by the minimum size of an individual [47].

Whilst gregarious behaviour is an important aspect of settlement and recruitment of many barnacle species, including *A* *. modestus* [48], no obvious gregarious recruitment patterns were observed at the relatively small spatial scales tested in this study (Knights pers. obs). Gregarious behaviour is a result of chemical cues that can be short lived in the field and can be initiated in response to the presence of other barnacle species [49]. Considering the length of the sampling period, the high levels of recruitment to the statues, and the lack of any other barnacle species, any initial behavioural responses may have been obscured. Additionally, Knight-Jones [48] found that during intense periods of settlement of *A* *. modestus* the distribution of barnacles on to settlement plates was even, in contrast to gregarious clumping during periods of light settlement. Despite high post-settlement mortality and an apparent lack of aggregated settlement behaviour, recruit densities on the statues remained high (~40 individuals cm^-2^) and more than double those reported on natural habitats [50]. Artificial structures are often characterised (especially when first installed) by opportunistic species, low species richness, an absence of predators and assemblages markedly different to those on natural shores [32] where higher species diversity, competition and predation can infer natural resistance to invasion [6]. The absence of a diverse or established community can lead to artificial structures being dominated by introduced species [8,51] and here this was illustrated by dense monocultures of *A* *. modestus* .

Artificial structures have been implicated as vectors of non-native species spread especially following disturbance (e.g. [26,52]). Clearly, invasive species can colonise disturbed areas quickly as shown by the rapid expansion of *A* *. modestus* throughout the UK and Europe [34] and the rate at which monocultures of *A* *. modestus* were re-established following their removal in this study. Given that artificial structures are likely to continue to proliferate and that current climate change projections suggest greater disturbance, free-space is likely to become available more frequently in the future. Correlations between disturbance and invasion success are becoming more widely documented (e.g. [5,53]) and suggest that the abundance of native species is likely to decline, although these relationships are far from straightforward [5]. Under these conditions, highly competitive and fast-colonising invasive species, such as *A* *. modestus* , are expected to increase in both abundance and distribution and the proliferation of artificial structures could act to support their expansion.
